# Associations of Genetic Polymorphisms and Neuroimmune Markers With Some Parameters of Frontal Lobe Dysfunction in Schizophrenia

**DOI:** 10.3389/fpsyt.2021.655178

**Published:** 2021-05-07

**Authors:** Anna Morozova, Yana Zorkina, Konstantine Pavlov, Olga Pavlova, Olga Abramova, Valeria Ushakova, Alexander V. Mudrak, Svetlana Zozulya, Irina Otman, Zoya Sarmanova, Tatiana Klyushnik, Alexander Reznik, Georgiy Kostyuk, Vladimir Chekhonin

**Affiliations:** ^1^Department Basic and Applied Neurobiology, V. P. Serbsky Federal Medical Research Centre of Psychiatry and Narcology, Moscow, Russia; ^2^Mental-Health Clinic No. 1 Named After N. A. Alexeev of Moscow Healthcare Department, Moscow, Russia; ^3^Department of Biology, Lomonosov Moscow State University, Moscow, Russia; ^4^Mental Health Research Center, Moscow, Russia; ^5^Institute of Medical and Social Technologies, Moscow, Russia; ^6^Department of Medical Nanobiotechnology, Pirogov Russian National Research Medical University, Moscow, Russia

**Keywords:** schizophrenia, PANSS, FAB, *DRD3*, BDNF, *HTR2A*, leukocyte elastase, α1-proteinase inhibitor

## Abstract

We investigated the associations of *DRD3* rs6280, *HTR1A* rs6295, *BDNF* rs6265, *SCL6A4* rs16965628, and *5HT2A* rs7322347 with schizophrenia in a case–control study, and associations of these genetic variants with several clinical features. We also investigated markers of inflammatory response (C-reactive protein, IL-2, IL-6, IL-10), the activity of leukocytic elastase (LE) and α1-proteinase inhibitor (a1-PI), antibodies to S100B and myelin basic protein (MBP) in schizophrenia. Clinical symptoms were assessed on three scales: Positive and Negative Syndrome Scale, The Bush – Francis Catatonia Rating Scale and Frontal Assessment Battery. All SNPs were typed using predesigned TaqMan SNP genotyping assays. The biomarkers related to the immune system were routinely tested using ELISA kits. The association with schizophrenia was found for *DRD3* rs6280 (*p* = 0.05) and *HTR2A* rs7322347 (*p* = 0.0013). We found differences between groups by parameters of LE and a1-PI and LE/a1-PI (*p* < 0.001). And IL-6 was evaluated in the schizophrenia group (*p* < 0.001). We showed that patients with the TT allele (*BDNF* rs6265) had more severe impairments in frontal lobe function. a1-PI can serve as a marker for assessing the severity of frontal lobe damage in patients with frontal dementia. We found some biological parameters reflecting the severity of frontal dysfunction in schizophrenia.

## Introduction

Schizophrenia spectrum disorders are severe and debilitating psychiatric disorders hereditary in nature, mostly chronic. They have a heterogeneous neurobiological background affecting brain development. Schizophrenia is a multifactorial disease, where both hereditary, development and environmental risks factors are involved (1).

Due to diverse clinical phenotypes and heterogeneity of the overlapping symptoms and functional deficits in schizophrenia spectrum disorders, their biological background is still not fully understood. A large number of hypotheses regarding the pathogenesis of psychotic disorders is known nowadays. For a long time, it has been known that genetic background is a crucial factor of schizophrenia. The rationale behind genome-wide association study (GWAS) is the fact that schizophrenia is mainly associated with common genetic variants (SNPs). Large-scale GWAS have identified about 150 risk loci. Most of these common alleles confer only relatively with very small effect (typically odds ratios <1.2) but cumulatively they have been estimated to explain between a quarter and half of the variance in genetic liability (2). Besides, not all of them are functional (or non-synonymous) SNPs that have a deleterious effect on protein function.

We focused on SNPs of some genes, related to impaired metabolism of neurotransmitter systems in schizophrenia and selected the non-synonymous polymorphisms of *DRD3* (dopamine receptor type 3) rs6280, *HTR1A* (5-Hydroxytryptamine receptor type 1A) rs6295*, BDNF* (brain derived neurotrophic factor) rs6265 and *5HT2A* (5-Hydroxytryptamine receptor type 2A) rs7322347 for further investigation. To date, the largest GWAS of schizophrenia (3, 4) showed that selected SNPs are not reaching genome-wide statistical threshold (α = 5 × 10–8), but nominally are associated with the disease according to numerous studies (5–7).

The possible functional effect of the functional missense mutation in the *DRD3* gene (Ser9Gly, rs6280) -polymorphism on dopamine release is consistent with previous work demonstrating that the glycine allele yields D3 autoreceptors that have a higher affinity for dopamine and display more robust intracellular signaling (8, 9).

*5HT1A* SNP rs6295 (C-1019G) is located in the promoter region and is known to be a functional polymorphism that regulates *HTR1A* transcription and region-specific modification of *HTR1A* expression.

Previous studies identified the association between the rs6295 polymorphism and symptomatic improvement in schizophrenia during treatment with antipsychotics. In particular, the researches showed a significantly greater negative symptoms improvement than *G* allele (10) carriers and improved attention (11). Meta-analysis of five studies, including the three with lurasidone, demonstrated the association of rs6295 with improvement of positive and negative symptoms in patients with schizophrenia (12).

The involvement of the *HTR2A* in psychosis spectrum disorder was proved by post mortem studies reported a decreased density of serotonin 5-HT2 receptors in the prefrontal cortex, decreased level of *HTR2A* in the left planum temporale (sensory speech cortex) in schizophrenia (13, 14). The role of *HTR2A* in psychosis has also been investigated in gene association studies (13, 15). Thus, the *5HT2A* rs7322347 (A/T) was also associated with early-onset schizophrenia (16).

*BDNF* rs6265 (*Val66Met*), a non-synonymous substitution at codon 66: C → T/Valine → Methionine, is the most studied SNP in psychiatric genetics. The functional polymorphism rs6265 produces a profound effect on BDNF cellular biology. This SNP has been linked to BDNF subcellular trafficking.

The effects of the *BDNF Val66Met* polymorphism on cognition were extensively documented in schizophrenia and healthy individuals. The *Met* allele was associated with impairments in attentional processing, visuospatial ability and cognitive inflexibility in patients diagnosed with schizophrenia (17). This polymorphism was also associated with depressive symptoms in schizophrenia (18), severe positive symptoms, such as delusions (19) and hallucinations (20), cognitive impairments, and increased risk for schizophrenia (21).

5-HTT is encoded by the *SLC6A4* (serotonin transporter) gene. Serotonin transporter protein is primarily responsible for the termination of serotonin neurotransmission. The changes in serotonin transporter protein levels alter the amounts of serotonin receptors, serotonin synthesis and metabolism (22). SNP rs16965628, located on the intron of the *SLC6A4* is associated with suicides among patients with schizophrenia. Changes caused by rs1696528 C alleles may lead to alterations in the serotonergic system (23). Moreover, this allele is a candidate polymorphic region for etiology of obsessive-compulsive behavior (24).

Obviously, schizophrenia is a neurodevelopmental disorder with multiple biochemical abnormalities. The earliest hypothesis regarding the development of schizophrenia describes gene-controlled deficiencies in neurotransmitter metabolism (dopamine, serotonin, glutamate, gamma aminobutyric acid, and others) (25).

Later, there appeared a hypothesis that the neuroplasticity and neurotrophic function was a core of disturbances in some brain areas supported by cognitive deficits due to deterioration in central nervous system (CNS) and gradual progressive alterations in gray matter, occurring in neonatal period and adolescence was presupposed as possible mechanism. Neurotrophic factors were proposed as targets to reflect the degree of neurodamage (26).

Recently the role of inflammatory processes causing alterations of brain tissue and CNS functioning has also been considered as explanation to schizophrenia development, this is why immune status is an important indicator of course of schizophrenia. For psychotic disorders complex immune–brain interactions that affect neural development, survival, and functioning could play a substantial role (1, 27). High concentrations of the circulating proinflammatory cytokines and other immune markers are reported to be associated with increased risk of subsequent psychosis (27).

The given evidence suggests that schizophrenia is accompanied by changes in pro-inflammatory cytokines and other inflammatory markers in the brain (neuroinflammation) and the activation of the inflammatory responses in blood (28). Changes in cytokine profiles in schizophrenic patients imply immune and neurotransmission dysregulation (29). IL-2, IL-6, IL-10 are known markers of immune status in schizophrenia; and the alterations of their levels are connected with the severity of clinical symptoms and potential clinical implications. IL-2, IL-6 are increased and IL-10 is decreased in the first-episode psychosis vs. controls and in acutely ill patients with chronic schizophrenia vs. controls (27). Moreover, IL-6 concentrations are positively associated with both positive and negative symptoms, as assessed on the Positive and Negative Syndrome Scale (PANSS) (30).

There is also an evidence for associations between higher blood IL-6 and C-reactive protein (CRP) levels and cognition in patients with chronic and first-episode schizophrenia, including executive function, verbal and working memory, learning, and psychomotor speed, although these associations are not always replicable (31, 32).

Immunological markers of leukocyte (neutrophil) elastase (LE), α1-proteinase inhibitor (α1-PI) activities, and autoantibodies' levels against S-100-beta and myelin basic protein (MBP) were chosen because of their association with patients' clinical state assessed by PANSS. The significant correlations were found between the severity of patients' psychosis and the activity of LE in the blood, as well as anti-S100B antibodies (33, 34).

LE is a proteolytic enzyme of neutrophils that hydrolyzes proteins of blood plasma, including coagulation factors, fibrinolysis, kallikreinkinin system and complement. LE can be a destructive factor in relation to the blood-brain barrier permeability (35). The biological role of LE and its contribution to brain pathology was reviewed by Shimakura et al. (36). a1-proteinase inhibitor is the acute phase protein that regulates the proteolytic activity of LE and other proteinases and also provides conditions for limiting the source of inflammation and destruction (37). The ratio of the activity of LE and its inhibitor can be predictors of tissue damage in the brain determining the outcome of the inflammatory response (38). An increase in the level of antibodies to S100B and MBP characterize more severe course of the schizophrenic process (39).

It is known that cognitive dysfunctions and abnormalities in the underlying brain processes are considered, respectively, as core clinical symptoms and biological features in the pathophysiology of psychiatric disorders.

Studies during the past 20 years have found complex interactions between the immune system, systemic inflammation and the brain, leading to changes in mood, cognition, and behavior (1).

Cognitive impairments in schizophrenia are associated with neuroinflammatory markers such as CRP, S100B and neuron-specific enolase (40, 41), and with lower concentrations of BDNF (40).

The aim of this study was to research the associations between symptomatology and SNP of genes related to the systems of neurotransmission (*DRD3* rs6280, *HTR1A* rs6295, *5HT2A* rs7322347) and neurotrophic factor (*BDNF* rs6265) in schizophrenia patients. Also we investigated associations between some immunological patterns and markers of inflammatory response (CRP, IL-2, IL-6, IL-10, the activity of LE and a1-PI, antibodies to S100B and MBP) with clinical symptoms in schizophrenia.

## Materials and Methods

Patients (SZ) (*n* = 655, male-345, female-310) were recruited from Mental-health Clinic No. 1 named after N.A. Alekseev of Moscow Healthcare Department, Moscow (Russia). The diagnoses included in the study were (F20 – 627 patients) Schizophrenia, (F22 – 3 patients) Persistent delusional disorders, (F23 – 8 patients) Acute and transient psychotic disorders, (F25 – 17 patients) Schizoaffective disorders. The average age of patients was 33.6 ± 9.8 in women and 30.1 ± 7.9 in men. The average age of onset was 24.6 ± 8.1 in women and 22.3 ± 5.7 in men. The average total PANSS was determined to be 105 ± 24 for women and 109 ± 22 for men.

Healthy controls (HC) (*n* = 768) were recruited from the same geographical area as SZ group. All participants gave written informed consent for participation in the project.

Exclusion criteria for patients were age <18 years, serious medical or surgical illness, previous episode of psychosis due to substance abuse, and psychotic symptomatology within a clearly diagnosed affective or borderline personality disorder. For healthy controls, exclusion criteria were age <18 years, current or past psychiatric disorder, family history of any psychiatric disorder, head trauma, neurological illness, serious medical or surgical illness, or substance abuse. HC and SZ had no addictions including <80 mg in ethanol equivalent per week and <10 cigarettes per day.

SZ status was determined based on the International Classification of Disease (ICD-10). Patients were evaluated using a structured interview for the Positive and Negative Syndrome Scale (PANSS), including the PANSS positive, PANSS negative, and PANSS general psychopathology subscales, Frontal Assessment Battery (FAB), The Bush – Francis Catatonia Rating Scale (BFCRS). The Russian version of PANSS, with acceptable validity and reliability, was used to evaluate the severity of psychosis symptoms of schizophrenia patients in this study.

The protocol of this study was approved by the Interdisciplinary Ethics Committee, Moscow (22/07/2017). All data is available from the authors on request.

The antipsychotics used were those with the highest clinical response in each individual patient, medication status was described in previous article (42). We also added treatment information to Table 1 [Chlorpromazine equivalents are taken from the work Gardner et al. (43)].

**Table 1:** Baseline characteristics of patients and healthy control.

	**SZ genotype**		**HC genotype**		**SZ immunology**		**HC immunology**	
	**Male**	**Female**	**Male**	**Female**	**Male**	**Female**	**Male**	**Female**
Gender	345	310	645	123	189	174	78	72
Age	30.1 ± 7.9	33.6 ± 9.8	22.1 ± 5.5	24.6 ± 6.3	29.3 ± 5.9	34.4 ± 8.5	31.1 ± 6.7	33.8 ± 8.5
Education (higher)	65	102	153	58	37	57	14	30
Marriage (+)	59	136	97	35	32	76	10	25
Working	79	104	302	32	46	58	64	49
Student	37	33	323	86	18	18	12	15
Unemployed	229	173	20	5	125	98	2	8
Body Mass Index (kg/m^2^)	24 ± 3.9	23.5 ± 13.7	20 ± 4.3	21.1 ± 6.5	23 ± 5.9	23 ± 8.7	25 ± 7.9	24.1 ± 7.9
Never smoked	201	248	521	109	105	138	50	61
Stopped smoking	17	4	9	2	9	2	5	3
Smoking	127	58	115	12	75	34	23	8
F20	333	294			189	174		
F22	3	0			0	0		
F23	2	6			0	0		
F25	7	10			0	0		
Manifested age	22.3 ± 5.7	24.6 ± 8.1			23.8 ± 4.9	24 ± 8.6		
PANSS	109 ± 22	105 ± 24			99 ± 18	97 ± 19		
FAB	13 ± 5.3	13.4 ± 4.8			14 ± 4.6	14 ± 5		
BFCRS	4 (2;11)	3 (0; 12)			3 (1;10)	2 (0; 9)		
Average dose of antipsychotics, in chlorpromazine equivalent	813.6 ± 445.8	784.5 ± 393.1			786.6 ± 413.9	765.5 ± 403.2		

*F20, Schizophrenia; F22, Persistent delusional disorders; F23, Acute and transient psychotic disorders; F25, Schizoaffective disorders; PANSS, Positive and Negative Syndrome Scale; FAB, Frontal Assessment Battery; BFCRS, The Bush – Francis Catatonia Rating Scale*.

We took samples from patients in the hospital who needed urgent drug treatment. They mostly receive such treatment as early as the first aid stage before admission to the hospital. Therefore, it was difficult to completely exclude antipsychotic medication in our study. For our research we always take blood within 3–4 days of the patient's admission to hospital. A total of 10 ml fasting blood was collected from each subject by venipuncture in the morning before breakfast. Five milliliter was introduced into a free-anticoagulant vacuum tube and immediately centrifuged at 2,000 × g for 10 min. Five milliliter was placed into an ethylenediaminetetraacetic acid (EDTA) vacuum tube for DNA analysis. Serum and total blood were stored at −80°C for further analysis.

### Genotyping

Genomic DNA samples were obtained from peripheral blood lymphocytes using an automatic DNA extraction (QIAGEN QIAcube) system, according to the manufacturer's recommendations. DNA concentration and sample quality were assessed spectrophotometrically (NanoVue, GE Healthcare). The obtained DNA samples were normalized in TE buffer to a final concentration of 4 ng/μl in the 384-well plate format. All SNPs were typed using predesigned TaqMan SNP genotyping assays (Applied Biosystems, Thermo Fisher, USA). Assays ID: C__32472275_10, C__11904666_10, C__11592758_10, C____949770_20, C__29706403_10. PCR and allelic discrimination assays were run using a QuantStudio 5 Real-Time PCR system (Applied Biosystems, Thermo Fisher, USA).

### Immunological status

Immunological parameters were assessed in serum from blood collected from 363 patients and 150 healthy controls. Samples were stored at −20°C for 1–2 weeks before assays. LE activity (a measure of neutrophil degranulation activity) was assayed by an enzymatic method using the chromogenic substrate specific N-tert-butoxy-carbonyl-alanine-β-nitrophenyl ester (BOC-Ala-ONp) (Sigma and was expressed in nmol/min × ml. The LE-α1-PI complex was dissociated by adding acetonitrile to each serum sample (44). α1-PI functional activity was assayed spectrophotometrically and was determined based on its ability to inhibit esterase activity of trypsin, using Nα-Benzoyl-L-arginine ethyl ester (BAEE) as a substrate. The measurements were performed using an Ultrospec 1100 spectrophotometer (Amercham) and expressed in IU/ml (45). Antibodies to S-100-beta protein and myelin basic protein were assayed using an immunoenzyme assay (46). S-100B protein from bovine brain (product No. S6677, Sigma-Aldrich, USA) and myelin basic protein bovine (product No. M1891-1MG) were used for ELISA. The proteins were dissolved in deionized water at 1 mg/ml. Antibody titers were expressed in optical density units (OD) and each sample was assayed in duplicate. C-reactive protein (CRP) (product No. A-9002 CRP-EIA-BEST highly sensitive) was assayed using a solid-phase immunoenzyme assay using a reagent kit (Vector-Best, Novosibirsk, Russia) and was expressed in IU/l. IL-2, IL-6, and IL-10 were determined using the diagnostic kits for ELISA (product No. A-8772 Interleukin-2-EIA-BEST, product No. A-8768 Interleukin-6-EIA-BEST, product No. A-8774 Interleukin-10-EIA-BEST, Vector-Best, Novosibirsk, Russia) and were expressed in pg/ml. These kits are not evaluated the reference proteins.

### Statistical Analysis

All parameters were checked for normal distribution using a Shapiro-Wilk test. The association between genotype or immunological markers and clinical characteristics was evaluated using ANOVA Fisher test. To compare the genetic variations in the case-control study, the chi-square test was used. Descriptive statistics of neuroinflammatory biomarkers in the group of patients and healthy control was presented median with low and upper quartile (Me, LQ, UQ) because of non-normal distribution. For comparison with immunological marker controls, the Kruskal-Wallis test was used. Dispersion analysis of association of immunological markers with parameters of frontal dysfunction on the FAB, in the group of patients with signs of frontal dementia was evaluated using ANOVA Fisher test. The classification of objects (the values of immunological markers) based on the distances between them was carried out by two-step cluster analysis with first standardization of variables. Bonferroni and FDR- corrections were applied to correct the *p*-values to control inflation of the type I error rate. The differences were considered significant at *p* < 0.05. Statistical analyses were performed with the R program (2016 The R Foundation) and IBM SPSS Statistics 26.

## Results

Table 1 depicts the demographic profile of the patients and healthy subject at baseline. No differences were found between patients and controls according to age, gender, and ethnicity.

Comparison of allele distribution frequencies of the investigated SNPs revealed significant differences for *DRD3* rs6280 (*p* = 0.05) and *HTR2A* rs7322347 (*p* = 0.0013) genes (Table 2). Comparison of allele frequencies in the case-control study: for *SCL6A4* rs16965628 chi-squared = 0.3, *p*-value = 0.86; for *HTR1A* rs6295 chi -squared = 3.5, *p*-value = 0.18; for *BDNF* rs6265 chi -squared = 0.9, *p*-value = 0.64; for *DRD3* rs6280 chi -squared = 5.92, *p*-value = 0.05; for *HTR2A* rs7322347 chi -squared = 13.3, *p*-value = 0.0013.

**Table 2:** Allelic distribution in SZ group and HC group.

		**HC**				**SZ**			
		**Male**	**HWE**	**Female**	**HWE**	**Male**	**HWE**	**Female**	**HWE**
SCL6A4 rs16965628	C/C	6 (1%)	Minor AF = 0.07	0 (0%)	Minor AF = 0.08	7 (2%)	Minor AF = 0.06	0 (0%)	Minor AF = 0.06
	C/G	77 (12%)	χ^2^ = 3.2	17 (14%)	χ^2^ = 0.68	28 (8%)	χ^2^ = 29.2	40 (13%)	χ^2^ = 1.47
	G/G	561 (87%)	*p* = 0.11	106 (86%)	*p* = 0.9	311 (90%)	*p* < 0.01	270 (87%)	*p* < 0.01
HTR1A rs6295	C/C	135 (21%)	Minor AF = 0.48	44 (36%)	Minor AF = 0.41	79 (23%)	Minor AF = 0.48	68 (22%)	Minor AF = 0.47
	C/G	355 (55%)	χ^2^ = 6.7	55 (45%)	χ^2^ = 0.61	204 (59%)	χ^2^ = 11.9	189 (61%)	χ^2^ = 15.3
	G/G	155 (24%)	*p* = 0.01	23 (19%)	*p* = 0.46	62 (18%)	*p* < 0.01	53 (17%)	*p* < 0.01
BDNF rs6265	C/C	477 (74%)	Minor AF = 0.13	89 (72%)	Minor AF = 0.16	245 (71%)	Minor AF = 0.16	214 (69%)	Minor AF = 0.17
	C/T	161 (25%)	χ^2^ = 3.62	27 (22%)	χ^2^ = 5.4	93 (27%)	χ^2^ = 0.28	84 (27%)	χ^2^ = 1.05
	T/T	6 (1%)	*p* = 0.06	7 (6%)	*p* = 0.05	7 (2%)	*p* = 0.69	12 (4%)	*p* = 0.32
DRD3 rs6280	C/C	26 (4%)	Minor AF = 0.23	0 (0%)	Minor AF = 0.19	21 (6%)	Minor AF = 0.25	28 (9%)	Minor AF = 0.28
	C/T	245 (38%)	χ^2^ = 3.31	48 (39%)	χ^2^ = 7.2	135 (39%)	χ^2^ = 0.21	118 (38%)	χ^2^ = 2.38
	T/T	374 (58%)	*p* = 0.076	75 (61%)	*p* = 0.01	190 (55%)	*p* = 0.78	164 (53%)	*p* = 0.32
HTR2A rs7322347	A/A	45 (7%)	Minor AF = 0.3	21 (17%)	Minor AF = 0.35	48 (14%)	Minor AF = 0.37	65 (21%)	Minor AF = 0.3
	A/T	310 (48%)	χ^2^ = 9.8	44 (36%)	χ^2^ = 5.6	159 (46%)	χ^2^ = 0.04	136 (44%)	χ^2^ = 3.4
	T/T	290 (45%)	*p* = 0.002	58 (47%)	*p* = 0.02	138 (40%)	*p* = 0.82	109 (35%)	*p* = 0.065

*The table shows the number of patients with each genotype, and the percentage in brackets. HWE, Hardy-Weinberg Equilibrium; Minor AF, Frequence of minor allele; HC, healthy control; SZ, schizophrenia; χ^2^, Chi-Square Tests for Hardy-Weinberg Equilibrium; p, p-value. P-values for HWE were calculated by Chi square test with one degree of freedom (df -1)*.

During the analysis of SNPs relationship with PANSS we did not find significant associations. Nevertheless, we detected the associations with parameters of BFCRS: Waxy flexibility (*F* = 4.83 *p* = 0.0085), Negativism (*F* = 4.28 *p* = 0.01), Stupor (*F* = 5.9 *p* = 0.003), Catalepsy (*F* = 5.63 *p* = 0.004). Patients with genotype T/T (*Met66Met* polymorphism) demonstrated higher scores, which reflect more severe symptoms. Additionally, we observed that these symptoms were not related to the age of the patient.

We also found associations between *BDNF* rs6265 and FAB. In particular, patients with genotype T/T (Met66Met) showed lower FAB scores (*p* = 0.011 for female and 0.0018 for male). Statistical differences were also demonstrated for all parameters of this scale: 1. Similarities (*F* = 3.3 *p* = 0.038); 2. Lexical Fluency (*F* = 5.04 *p* = 0.007); 3. Motor Series (*F* = 3.96 *p* = 0.019); 4. Conflicting Instructions (*F* = 4.16 *p* = 0.0016); 5. Go–No Go (*F* = 2.14 *p* = 0.12); 6. Prehension Behavior (did not pass Bonferroni correction, *F* = 4.11 *p* = 0.017).

We did not observe any differences between SZ and HC in terms of parameters IL-10, CRP, antibodies to S100B and MBP (Table 3). We detected the statistical differences between groups by parameters of the activity of LE and a1-PI, and LE/a1-PI (*p* < 0.001). IL-6 was evaluated in both male and female in comparison to HC groups (*p* < 0.001) (Table 3).

**Table 3:** Descriptive statistics of neuroinflammatory biomarkers in the group of patients and healthy control.

		**Male**	**Kruskal-Wallis test;** ***p*-value**	**Female**	**Kruskal-Wallis test;** ***p*-value**
LE	Patients	239.75 (213.8; 267.8)	**KW = 24.5**	234 (211.7; 270.55)	**KW = 15.91**
	Control	208.9 (197.6; 222.1)	***p*** **< 0.001**	216 (212.15; 217.45)	***p*** **< 0.001**
a1-PI	Patients	45.9 (38.4; 53.4)	**KW = 41.67**	45.9 (38.5; 51.52)	**KW = 31.4**
	Control	34.4 (31.25; 37.35)	***p*** **< 0.001**	32.6 (30.9; 33.05)	***p*** **< 0.001**
a-S100B	Patients	0.71 (0.65; 0.78)	KW = 0.69	0.76 (0.68; 0.88)	KW = 1.02
	Control	0.71 (0.63; 0.8)	*p* = 0.4	0.77 (0.73; 0.8)	*p* = 0.31
a-MBP	Patients	0.73 (0.64; 0.83)	KW = 0.1	0.77 (0.7; 0.91)	KW = 0.5
	Control	0.74 (0.65; 0.85)	*p* = 0.75	0.81 (0.77; 0.86)	*p* = 0.48
LEa1	Patients	5.31 (4.47; 6.29)	**KW = 10.25**	5.39 (4.35; 6.6)	**KW = 9.41**
	Control	6.15 (5.71; 6.66)	***p*** **= 0.0014**	6.58 (6.53; 6.79)	***p*** **= 0.0022**
IL-6	Patients	3.48 (1.72; 4.55)	**KW = 13.34**	3.62 (2.76; 4.67)	**KW = 8.57**
	Control	1.98 (1.84; 2.27)	***p*** **< 0.001**	2.1 (1.86; 2.24)	***p*** **< 0.001**
IL-10	Patients	6.2 (2.96; 11.4)	KW = 0.017	6.1 (2.88; 11.25)	KW = 1.7
	Control	7.74 (6.2; 9.23)	*p* = 0.9	8.98 (8.22; 9.31)	*p* = 0.19
CRP	Patients	1.74 (0.71; 5.76)	KW = 3.58	1.25 (0.36; 3.81)	KW = 1.51
	Control	0.84 (0.441.64;)	*p* = 0.058	0.63 (0.37; 1.44)	*p* = 0.22

*Data are presented as median (Me), low and upper quartile (LQ, UQ), LE, leukocyte (neutrophil) elastase; a1-PI, α1-proteinase inhibitor; a-S100B, autoantibodies against S-100-beta; MBP, myelin basic protein; LEa1, leukocyte elastase/α1-proteinase inhibitor ratio; IL-6, interleukin-6; IL-10, interleukin-10; CRP, C-reactive protein*.

The correlation between the activity of LE and a1-PI, a-S100B, and a-MBP and the total score of the PANSS scale (including the subscale of positive, negative symptoms and subscale of general psychopathology), NSA, BFCRS did not reveal statistically significant differences.

Analysis of associations of LE and a1-PI activity, a-S100B, a-MBP with a total FAB score, did not reveal significant differences with a1-PI (*F* = 1.045, *p* = 0.31), a-S100B (*F* = 0.002, *p* = 0.96), a-MBP (*F* = 0.491, *p* = 0.48).

For the activity of LE this dependence was demonstrated with *F* = 6.018 *p* = 0.0148, but this result did not pass for multiple comparisons.

The considerable variability for each of the analyzed markers in the total group of patients served as the basis for cluster analysis. Three immunological subgroups differing in the activity of LE, LE/a1-PI, and the level of autoimmune reactions in comparison to the HC group were revealed. A significant increase in the activity of LE in all groups compared to controls was shown (*p* < 0.001). The differences in profiles of immunological markers in subgroups of schizophrenia patients reflect the different types of the inflammatory response to pathological process (Table 4).

**Table 4:** Descriptive statistics of neuroinflammatory biomarkers in subgroups of patients and healthy control.

		**Immunological subgroups, Me (LQ;UQ)**			**Kruskal-Wallis test; *p*-value**	**Immunological subgroups (U, Z, *p*-value)**
		**1**	**2**	**3**		
LE	Patients	285.1 (275.4; 299.2)	238.6 (228.4; 252.7)	205.2 (190.1; 211.7)	**231.4**	U^1−2^ = 0, Z = −11.82, ***p*** **< 0.001**
	Control	211.3 (198.75; 219.42)	211.3 (198.75; 219.42)	211.3 (198.75; 219.42)	**< 0.001**	U^2−3^ = 0, Z = −11.792, ***p*** **< 0.001**
						U^1−3^ = 0, Z = −10.902, ***p*** **< 0.001**
a1-PI	Patients	47.9 (39.6; 50.6)	43.9 (38.1; 52.5)	46.0 (38.0; 53.8)	1.73	U^1−2^ = 3,431, Z = −1.271, *p* = 0.204
	Control	33.85 (31.17; 37.07)	33.85 (31.17; 37.07)	33.85 (31.17; 37.07)	0.42	U^2−3^ = 4,017, Z = −0.971, *p* = 0.332
						U^1−3^ = 3,156.5, Z = −0.106, *p* = 0.916
a-S100B	Patients	0.77 (0.7; 0.85)	0.73 (0.65; 0.78)	0.74 (0.65; 0.81)	**6.42**	U^1−2^ = 3,013, Z = −2.504, ***p*** **= 0.012**
	Control	0.71 (0.64; 0.8)	0.71 (0.64; 0.8)	0.71 (0.64; 0.8)	**0.04**	U^2−3^ = 4,230, Z = −0.397, *p* = 0.691
						U^1−3^ = 2,649, Z = −1.842, *p* = 0.065
a-MBP	Patients	0.77 (0.7; 0.9)	0.74 (0.64; 0.89)	0.74 (0.64; 0.85)	**4.28**	U^1−2^ = 3,381.5, Z = −1.417, *p* = 0.156
	Control	0.75 (0.68; 0.85)	0.75 (0.68; 0.85)	0.75 (0.68; 0.85)	**0.12**	U^2−3^ = 4,134, Z = −0.656, *p* = 0.512
						U^1−3^ = 2,583, Z = −2.068, ***p*** **= 0.039**
LEa1	Patients	6.2 (5.4; 7.3)	5.4 (4.6; 6.5)	4.3 (3.7; 5.2)	**62.8**	U^1−2^ = 2,561, Z = −3.834, ***p*** **< 0.001**
	Control	6.26 (5.84; 6.68)	6.26 (5.84; 6.68)	6.26 (5.84; 6.68)	**< 0.001**	U^2−3^ = 2,383.5, Z = −5.37, ***p*** **< 0.001**
						U^1−3^ = 1,048.5, Z = −7.314, ***p*** **< 0.001**

*Data are presented as median (Me), low and upper quartile (LQ, UQ). Differences in neuroinflammatory markers in subgroups of patients and healthy control. Differences were evaluated using Kruskal-Wallis test. LE, leukocyte (neutrophil) elastase; a1-PI, α1-proteinase inhibitor; a-S100B, autoantibodies against S-100-beta; MBP, myelin basic protein; LEa1, leukocyte elastase/α1-proteinase inhibitor ratio*.

Analysis of associations of markers in immunological subgroups with the severity of clinical symptoms revealed no differences in the total FAB score and in the total BFCRS score. ANOVA between immunological subgroups for FAB *F* = 2.776, *p* = 0.064 and *F* = 2.793, *p* = 0.063 for BFCRS scale.

We showed that a subgroup of patients with signs of frontal dementia (11 or lower points on the FAB scale) (38 male, 39 female) significantly differs by immunological parameters from others. Moreover, these differences did not depend on the age of the patients. For them the dispersion analysis of the connection of these inflammation markers with the total FAB score and with each parameter of this scale (see Supplementary Materials) was carried out.

Most of patients with signs of frontal dementia on the FAB scale were distributed between 2 and 3 immunological subgroups. Pearson's chi-square test for clustered data in immunological subgroups and parameters of the FAB scale (%): for FAB score > 11: 32.7; 41.7; 25.6 for 1, 2, and 3 subgroups; for FAB score ≤ 11: 21.1; 34.7; 44.2 for 1, 2, and 3 subgroups. X-squared = 10.156, df = 2, *p*-value = 0.006.

No statistically significant associations between genotype and immune status parameters were found (data on request).

## Discussion

In the first part of our study, we demonstrated some associations between genetic polymorphism with cognitive and motor disturbances in patients with schizophrenia spectrum disorder and confirmed the involvement of certain genetic factors in symptomatology of the disease. In the second part of the study we verified the involvement of neuroimmunological biomarkers in schizophrenia spectrum disorders.

We have shown the associations of *HTR2A rs7322347* and *DRD3 rs6280* with schizophrenia spectrum disorder in the case-control study. This data confirms our earlier findings (16). Despite the fact that the associations of *BDNF rs6265, HTR1A rs6295, SCL6A4 rs16965628* with schizophrenia in case-control study was not found, we discovered interesting correlations between *BDNF rs6265* and some features of the disease. *Met66Met* polymorphism was associated with high scores of catalepsy, waxy flexibility, stupor and negativism – serious symptoms of catatonia measured by BFRS. These signs of motor abnormalities can indicate the severity of disease (47). Besides, it is known that the catatonia symptoms can reflect the extreme severity of affective deficit (48). According to other studies patients with schizophrenia accompanied by catatonic symptoms are found to have lower serum BDNF levels (49, 50), which proves the link between BDNF and catatonia symptoms (51). According to findings of other researcher, motor functions significantly correlate with cognitive function in patients with schizophrenia (52). In addition to the association of *Met66Met* with motor disturbance, we also found a correlation with frontal lobe dysfunction.

Patients with *Met66Met* had low scores of the FAB. Other studies also showed causality between Met allele of BDNF and cognitive disability (53–56). Kim et al. (57) demonstrated the association between *BDNF rs6265* and higher scores in PANSS scale and lower in cognitive tests in female patients with schizophrenia, but no performance difference was observed in male patients. In our study frontal lobe cognitive impairment was associated with *Met* allele in both male and female, regardless of their age.

In the second part of our research we have analyzed the serum CRP, IL-2, IL-6, IL-10 levels, the activity of LE and a1-PI, antibodies to S100B and MBP and their associations with clinical symptoms. We showed significant increase of IL-6 level in patients with schizophrenia spectrum disorder compared to healthy controls. Moreover, we demonstrated that the increase of serum activity of LE and a1-PI may be related to the diagnosis of schizophrenia, which confirms the data we obtained earlier (33) so as the other authors (58, 59). It has previously been demonstrated that patients with schizophrenia have lower serum BDNF, MBP, and GFAP levels, but higher serum IL-6 and S100B (58). The Ota 2015 study showed an increase of MBP gene expression in the peripheral blood of antipsychotic-naïve patients with first-episode psychosis (59).

The ratio of the activity of LE and its inhibitor characterizes the state of the leukocyte-inhibitory system and is considered as an unfavorable factor in the maintenance of tissue damage (38).

The differences of immunological profiles are associated with the different state of the leukocyte-inhibitory system of inflammation in schizophrenia.

As a result of cluster analysis, three immunological groups (clusters) associated with different state of leukocyte-inhibitory system in the response to the pathological process in brain were revealed.

The results are consistent with previously obtained data and indicate the involvement of the immune system (inflammation) in pathogenesis of schizophrenia. The state of leukocyte-inhibitory system of inflammation assessed by leukocyte-inhibitory index determines the course and the outcome of the inflammatory reaction. Pro-inflammatory potential associated with the increase in the activity of both LE and α1-PI compared to control group (*p* < 0.001 and *p* < 0.001) in patients of cluster 1 indicates the activation of systemic inflammatory reactions in the response to impaired homeostasis.

Two and 3 subgroups are characterized by an insufficient functional activity of neutrophils (by the activity of LE) and a significant increase of a1-PI activity (*p* < 0.05, *p* < 0.001, respectively). These profiles reflect a different degree of imbalance in the inflammatory response to impaired brain homeostasis.

Many studies indicate the assosiation between neural inflammation markers and cognitive functions (31, 32). In our study, cognitive functions were evaluated using the FAB, which indicates the degree of frontal dysfunction.

Bao demonstrated that serum levels of a1-PI in patients with chronic disorder of consciousness may have prognostic potential to disease severity (60). The prefrontal cortex has extensive mutual relations with wake-promoting centers in the brainstem and diencephalon, and therefore is in a unique position to modulate the level of consciousness. Thus, cholinergic mechanisms in the prefrontal cortex can regulate the level of consciousness (61). It has been shown that loss of consciousness after some brain injuries is associated with the reduced functional connectivity of the default mode network (DMN), fronto-parietal network, and thalamo-cortical network (62). Cognitive functions have been shown to be related to consciousness, suggesting that the neural correlates of memory formation and retrieval coincide with the neural correlates of conscious perception (63).

Presumably, the imbalance of leukocyte-inhibitory system associated with insufficient activity of LE (*p* < 0.05) and high activity of its inhibitor α1-PI (*p* < 0.001) in patients of cluster 3 may be due to functional depletion of neutrophils. This immune profile is associated with the severity of clinical symptoms in schizophrenia patients with frontal dysfunctions. Obtained results indicate that the imbalance of leukocyte-inhibitory system can be considered as a marker of the severe disease course and as an unfavorable prognostic factor. A similar immunological feature was previously identified in patients with catatonic disorders in schizophrenia (64).

Thus, the fact that the frontal lobe dysfunction altered consciousness, and data on a significant increase of this indicator activity accompanied by an insufficient increase of LE activity, as well as the correlation of a1-PI in blood serum with the severity of the consciousness disorder suggest that functional activity of this marker may serve as a potential prognostic factor for frontal function disorders in schizophrenia in our patients. According to the literature, patients with scores 11 and below have signs of frontal dementia. The group of patients with frontal dementia showed a decrease in the activity of a1-PI while increasing the score for each of the scale parameters. However, no such associations were found during the analysis of all patients. It was shown for the first time that activity of a1-PI can serve as a potential biomarker of cognitive deficit severity in the group of patients who demonstrates the signs of frontal dementia, but not in patients with normal frontal lobes function.

Despite the absence of differences in MBP serum concentration with healthy controls, in the group of patients with frontal dementia the inverse dependence of serum concentration on parameters such as 3. Motor Series (“Luria's Test”), 4. Conflicting Instructions, 5. Go-No Go (inhibitory control), 6. Prehension Behavior and FAB total score were found. We demonstrated that the serum activity of LE and a1-PI, and antibodies to the protein S100B and MBP may be related to the diagnosis of schizophrenia, which confirms the data obtained earlier by us (33).

The prefrontal cortex has extensive mutual relations with wake-promoting centers in the brainstem and diencephalon, and therefore is in a unique position to modulate the level of consciousness. Thus, cholinergic mechanisms in the prefrontal cortex can regulate the level of consciousness (61). It has been shown that loss of consciousness after some brain injuries is associated with the reduced functional connectivity of the default mode network (DMN), fronto-parietal network, and thalamo-cortical network (62).

## Conclusions

The feature of this study is the combined investigation of genotype and immune markers in the patients with schizophrenia spectrum disorder. As statistically significant associations between *Met* of *BDNF* gene and a1-PI and the degree of frontal dysfunction were demonstrated, these parameters can serve as markers for assessing the severity of frontal lobe damage in schizophrenia patients. Observed association between *Met* of *BDNF* gene and some severe signs of the motor dysfunction (catatonia signs) requires further study and verification.

## Data Availability Statement

The original contributions presented in the study are publicly available. This data can be found here: https://www.ebi.ac.uk/ena/browser/view/PRJEB42835.

## Ethics Statement

The study was conducted according to the guidelines of the Declaration of Helsinki, and approved by the Interdisciplinary Ethics Committee, Moscow (22/07/2017). The patients/participants provided their written informed consent to participate in this study.

## Author Contributions

AM, YZ, and SZ: writing—original draft preparation. OA and VU: writing—review and editing. KP and OP: methodology–genotyping. AVM, AR, and GK: methodology–patients. SZ, IO, and ZS: methodology–immune analysis. YZ and SZ: data analysis. GK and VC: supervision. AM and GK: project administration. GK: funding acquisition. All authors contributed to the article and approved the submitted version.

## Conflict of Interest

The authors declare that the research was conducted in the absence of any commercial or financial relationships that could be construed as a potential conflict of interest.

## Abbreviations

a1-PI: α1-proteinase inhibitor
a-S100B: autoantibodies against S-100-beta
BDNF: brain-derived neurotrophic factor
BFCRS: The Bush –Francis Catatonia Rating Scale
CNS: central nervous system
CRP: C-reactive protein
DRD3: dopamine 3 type receptor
FAB: Frontal Assessment Battery
GWAS: genome-wide association study
HTR1A: 5-Hydroxytryptamine Receptor 1A
HTR2A: 5-Hydroxytryptamine Receptor 2A
LE: leukocyte (neutrophil) elastase
MBP: myelin basic protein
PANSS: Positive and Negative Syndrome Scale
SLC6A4: serotonin transporter gene
SNPs: single-nucleotide polymorphisms.
