# Supplementary material for: Associations of Genetic Polymorphisms and Neuroimmune Markers With Some Parameters of Frontal Lobe Dysfunction in Schizophrenia
Source: Front Psychiatry. 2021 May 7;12:655178. doi: 10.3389/fpsyt.2021.655178 (PMC8138937; doi:10.3389/fpsyt.2021.655178)
Supplement: Supplementary file 1 [file Table_1.DOCX]

Dispersion analysis of association LE, a1, antibodies to the protein S100B, MBP with parameters of frontal dysfunction on the FAB scale, in the group of patients with signs of frontal dementia (scores on the scale 11 and less). Red color indicates associations that have passed FDR-correction for multiple comparison.

| **FAB** | **LE** | | **a1** | | **a-S100B** | | **a-MBP** | | **LE/a1** | |
| --- | --- | --- | --- | --- | --- | --- | --- | --- | --- | --- |
|  | **F** | **p-value** | **F** | **p-value** | **F** | **p-value** | **F** | **p-value** | **F** | **p-value** |
| Gender | 0,588 | 0,445 | 1,75 | 0,19 | 2,1 | 0,15 | 1,75 | 0,19 | 2,68 | 0,1 |
| 1. Similarities (conceptualization) | 1,546 | 0,219 | 4,44 | 0,0147 * | 2,97 | 0,0566 | 3,815 | 0,0259 * | 1,336 | 0,268 |
| *Gender | 0,508 | 0,604 | 0,34 | 0,7104 | 1,57 | 0,214 | 1,005 | 0,3704 | 1,115 | 0,333 |
| 2. Lexical Fluency | 0,934 | 0,428 | 3,19 | 0,028 * | 3,04 | 0,034 * | 0,886 | 0,452 | 1,267 | 0,291 |
| *Gender | 0,16 | 0,852 | 1,23 | 0,298 | 2,13 | 0,126 | 3,397 | 0,0382 * | 0,546 | 0,581 |
| 3. Motor Series ("Luria's Test") | 0,853 | 0,43 | 4,08 | 0,0204 * | 0,82 | 0,446 | 1,602 | 0,207 | 0,968 | 0,384 |
| *Gender | 0,049 | 0,952 | 0,208 | 0,813 | 0,91 | 0,407 | 4,885 | 0,0098 ** | 0,047 | 0,954 |
| 4. Conflicting Instructions | 1,215 | 0,309 | 4,36 | 0,006 ** | 3,12 | 0,03 * | 6,673 | 0,0004 *** | 1,186 | 0,32 |
| *Gender | 0,419 | 0,659 | 0,47 | 0,626 | 1,36 | 0,262 | 1,46 | 0,236 | 0,49 | 0,614 |
| 5. Go–No Go (inhibitory control) | 0,287 | 0,752 | 5,05 | 0,008 ** | 1,40 | 0,252 | 5,01 | 0,009 ** | 1,635 | 0,201 |
| *Gender | 1,454 | 0,239 | 1,05 | 0,355 | 2,485 | 0,089 | 1,431 | 0,245 | 0,042 | 0,959 |
| 6. Prehension Behaviour | 1,733 | 0,167 | 4,02 | 0,0101 * | 1,884 | 0,139 | 0,932 | 0,429 | 1,859 | 0,143 |
| *Gender | 0,603 | 0,615 | 0,5 | 0,68 | 0,464 | 0,708 | 2,878 | 0,041 * | 0,958 | 0,416 |
| FAB total score | 1,069 | 0,304 | 6,76 | 0,011 * | 1,642 | 0,203 | 7,96 | 0,006 ** | 1,571 | 0,213 |
| *Gender | 0,104 | 0,747 | 0,652 | 0,421 | 2,339 | 0,13 | 7,424 | 0,008 ** | 0,255 | 0,615 |
